# Disseminated Mucormycosis with Positive *Aspergillus* Galactomannan

**DOI:** 10.1155/2018/4294013

**Published:** 2018-12-24

**Authors:** Despoina Kitmiridou, Su N. Aung, Dimitrios Farmakiotis

**Affiliations:** ^1^Medical Student, National and Kapodistrian University of Athens, Athens, Greece; ^2^Division of Infectious Diseases, Warren Alpert Medical School of Brown University, Providence, USA

## Abstract

We describe a case of disseminated mucormycosis (*Apophysomyces elegans*) diagnosed on autopsy, in a man who had been working in construction with undiagnosed neutropenia from hairy-cell leukemia, which is rarely associated with invasive mold infections. Galactomannan values in both blood and bronchoalveolar lavage were strongly positive. There is an unmet need for accurate noninvasive fungal diagnostic tests. Detailed history, including occupational exposures, can be more informative than laboratory workup.

## 1. Case Presentation

A 48-year-old man was admitted to the intensive care unit with pneumonia and septic shock. He had no medical history, history of surgeries, or known allergies. He was not taking any medications. He worked as a pipefitter; otherwise, his social history was unremarkable. He had no significant family history. He tested positive for influenza B. He was treated with oseltamivir, vancomycin, piperacillin-tazobactam, and azithromycin. Chest X-ray showed patchy airspace disease in the right lung and focal consolidation in the left. Blood cultures returned positive for *Streptococcus pneumoniae*.

His absolute neutrophil count was 0, and peripheral flow cell cytometry showed hairy-cell leukemia, for which he received high-dose corticosteroids and rituximab.

**Figure 1:**
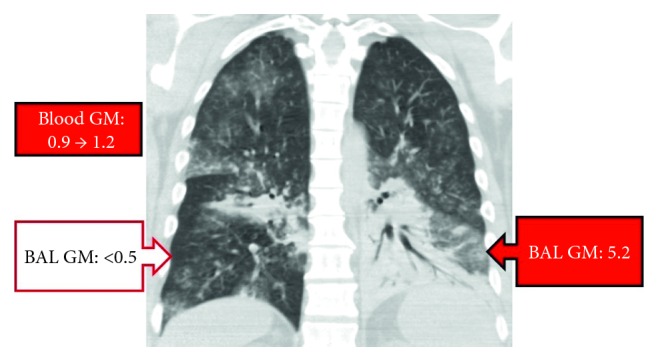
CT scan of the chest. Multifocal dense lung consolidations, most prominent in the left lower lobe, tree in bud opacities, and centrilobular nodules in the right lung; serum and left lower lobe: BAL galactomannan (GM) positive; right lower lobe: GM negative.

He developed anuric acute kidney failure requiring hemodialysis and marked elevation of liver function tests. On day 5, he had new fever, for which piperacillin/tazobactam was changed to meropenem. On day 9, blood cultures were positive for *Candida albicans*. Caspofungin was added. Chest CT revealed multifocal pneumonia. Bronchoscopy showed erythematous airways with minimal secretions. Bronchoalveolar lavage (BAL) galactomannan was strongly positive in the left lower lobe and negative in the right lower lobe. Serum galactomannan was positive x2 (Figure 1). BAL bacterial and fungal cultures were positive only for *C. albicans*.

**Figure 2:**
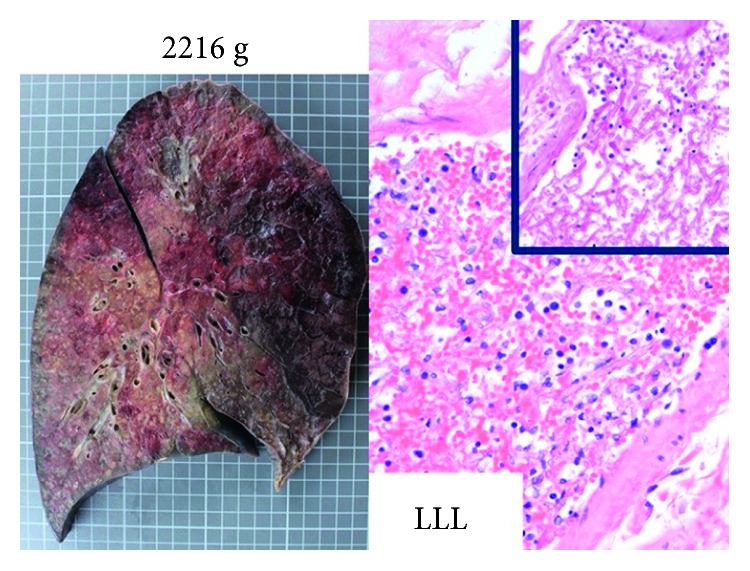
Postmortem autopsy findings revealed significantly congested lungs with infarction and necrosis. Histopathological hematoxylin-eosin stain showed abundant angioinvasive, aseptate hyphae, consistent with mucorales, in the left lower lung (LLL).

**Figure 3:**
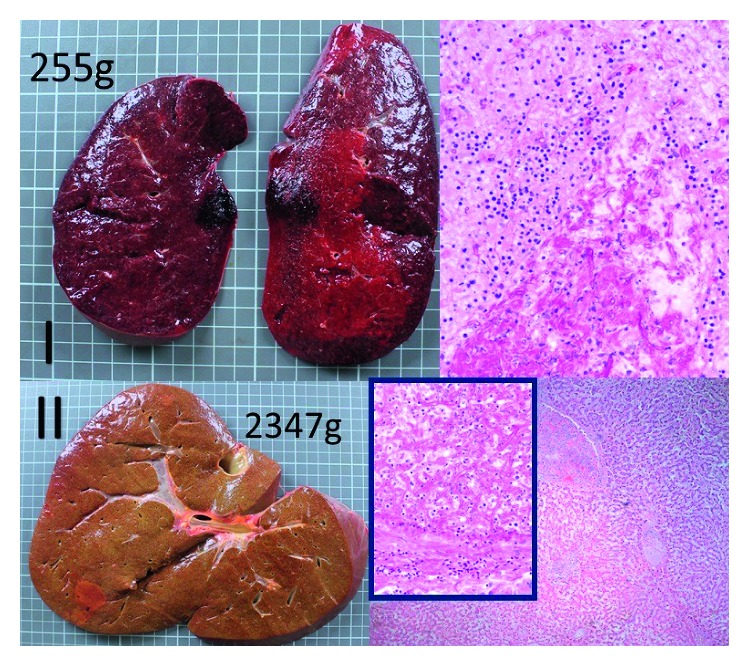
Postmortem autopsy findings revealed significant spleen (I) and liver (II) enlargement, congestion, infarction, and necrosis. Histopathological hematoxylin-eosin stain revealed abundant angioinvasive, aseptate hyphae, consistent with mucorales.

**Figure 4:**
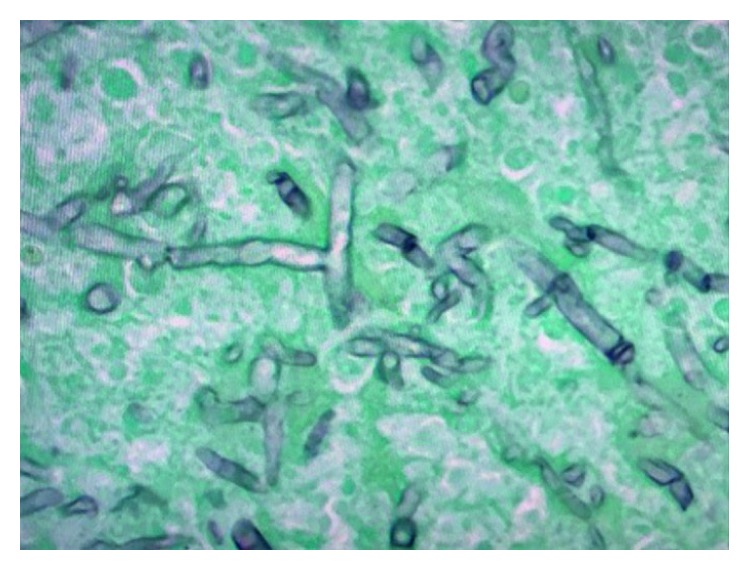
Gomori methenamine stain (GMS) showed abundant aseptate hyphae in the affected organs. The hyphal morphology (wide-angled, aseptate) was diagnostic of mucormycosis.

Isavuconazole was added on day 15 for probable invasive aspergillosis in the setting of multiorgan, including kidney (persistently anuric), and liver (bilirubin level of 15 mg/dL), and failure. He had massive hemoptysis and died one day after. Autopsy showed disseminated mucormycosis (Figures 2–4). Culture identified the species as *Apophysomyces elegans*.

## 2. Discussion

Although mucormycoses are rare, their frequency is likely underestimated. Most cases have been described in patients with poorly controlled diabetes, those treated with deferoxamine, and mainly in severely immunocompromised patients with history of solid organ or bone marrow transplant, or those neutropenic from acute myeloid or lymphoblastic, rather than hairy-cell leukemia [1]. Our patient, while neutropenic, likely inhaled through occupational exposure high inocula of *Apophysomyces* spores, which are abundant in soil [1, 2].

Disseminated mucormycosis usually results from progression of a localized (pulmonary) infection with hematogenous spread. It is frequently associated with nonspecific signs and symptoms, and almost always fatal [1]. In this case, high-dose corticosteroids might have accelerated fungal growth and dissemination.

Diagnosis of mucormycosis remains challenging and is often made by biopsy, because the yield of bronchoscopy is extremely low, since hyphae are angioinvasive in tissue, rather than the alveolar space [1]. Our case highlights these important limitations and the fact that galactomannan in blood or BAL has limited specificity and was likely false positive in this case [3]. The positive galactomannan could not have been caused by mucormycosis. In one study, significant *Candida* burden in the respiratory tract, which our patient had, was proposed as a potential cause of elevated BAL galactomannan [4]. Nevertheless, *Candid*a could not explain the positive serum galactomannan. Another possible explanation, which could not be entirely ruled out without molecular diagnosis, is a mixed mold infection with the mucorales outgrowing *Aspergillus*, since postinfluenza aspergillosis is a well-described clinical syndrome [5].

In mucormycoses, early initiation of amphotericin-B dramatically increases survival [1, 6]. The newer triazoles posaconazole and isavuconazole are active against the mucorales *in vitro* and in case series [1]. Although they are less toxic, their efficacy compared to amphotericin-B needs to be better defined.

## 3. Conclusions

Immunocompromised patients are at risk of multiple simultaneous life-threatening infections. Neutropenic patients in the community are at increased risk for invasive mold infection, especially when they have potential exposure to high inocula of spores. BAL cultures and fungal biomarkers may be misleading; thus, a detailed exposure and occupational history can be more informative. In high-risk immunocompromised patients, broad antimold prophylaxis or early empiric treatment should be considered. Novel noninvasive diagnostic modalities for invasive mold infections are urgently needed.
